# Attenuation of UVB-Induced Photo-Aging by Polyphenolic-Rich Spatholobus Suberectus Stem Extract Via Modulation of MAPK/AP-1/MMPs Signaling in Human Keratinocytes

**DOI:** 10.3390/nu11061341

**Published:** 2019-06-14

**Authors:** Kyoo-Ri Kwon, Md Badrul Alam, Ji-Hyun Park, Tae-Ho Kim, Sang-Han Lee

**Affiliations:** 1 Department of Food Science & Biotechnology, Kyungpook National University, Daegu 41566, Korea; rbfl0116@knu.ac.kr (K.-R.K.); mbalam@knu.ac.kr (M.B.A.); wlgus6744@knu.ac.kr (J.-H.P.); 2 Food and Bio-Industry Research Institute, Inner Beauty/Anti-ageing Center, Kyungpook National University, Daegu 41566, Korea; 3 Biomedical Research Institute, Kyungpook National University Hospital, Daegu 41944, Korea; kimth0929@ynu.ac.kr

**Keywords:** anti-aging, *Spatholobus suberectus*, matrix metalloproteinases (MMPs), collagen type I alpha 1 (COL1A1), elastin (ELN), mitogen-activated protein kinase (MAPK)

## Abstract

It is well known that ultraviolet light activates mitogen-activated protein (MAP) kinase by increasing the reactive oxygen species (ROS) in the body, enhancing activating protein 1(AP-1) complexes (c-Jun and c-Fos), increasing matrix metalloproteinases (MMPs) and degrading collagen and elastin. In this study, we confirmed that polyphenolic rich *Spatholobus suberectus* (SS) stem extracts suppressed ultraviolet (UV)-induced photo-aging. The major active components of SS stem extracts were identified as gallic acid, catechin, vanillic acid, syringic acid and epicatechin. The aqueous and ethanolic extracts of the stem of SS (SSW and SSE, respectively) significantly reduced the elastase enzyme activity. Moreover, both extracts were suppressed the ROS generation and cellular damage induced by UVB in HaCaT cells. Our results also revealed that SSE could regulate the expression of MMPs, tissue inhibitor of matrix metalloproteinase (TIMP)-1, collagen type I alpha 1 (COL1A1), elastin (ELN) and hyaluronan synthase 2 (HAS2) at their transcriptional and translational level. Furthermore, SSE was blocked the UVB-induced phosphorylation of mitogen-activated protein kinases (MAPKs), nuclear factor-kappa B (NF-κB) and c-Jun. Moreover, combination of syringic acid, epicatechin and vanillic acid showed strong synergistic effects on elastase inhibition activity, in which the combination index (CI) was 0.28. Overall, these results strongly suggest that the polyphenolics of SSE exert anti-ageing potential as a natural biomaterial to inhibit UVB-induced photo-aging.

## 1. Introduction

Skin aging is affected by many factors, such as ultraviolet radiation (UVR), oxidative stress, and inheritance. Among them, photoaging accounts for about 80% of skin aging [1]. UVR is divided into three wavelengths, including UVA (320–400 nm), UVB (290–320 nm), and UVC (200–290 nm). While UVC is absorbed by the ozone layer, UVA and UVB can reach the surface of the earth [2]. While most biomolecules cannot absorb UVA, UVB is predominantly injurious to living organisms. UVB contributes to detrimental effects directly through the production of reactive oxygen species (ROS) which is associated with DNA damage and inflection of gene expression. UVB initiates a photo-oxidation reaction primarily on the epidermis of the skin via augmentation of the cellular ROS level that causes imbalance of the skin antioxidant, sequentially accelerating photoaging [3,4]. UVB not only hinders collagen synthesis and promotes its breakdown, but also boosts the degradation of elastin in fibroblasts, resulting in the loss of skin elasticity which make deep wrinkle formation [5].

Collagen constitutes about 70% of the dermal layer and both matrix metalloproteinases (MMPs), and tissue inhibitors of matrix metalloproteinases (TIMPs) play a pivotal role in collagen degradation in epidermal keratinocytes and dermal fibroblasts. Up to now, 28 MMPs and 4 TIMPs have been identified [6]. MMPs are a family of zinc-dependent enzymes which are responsible for the degradation of collagen and extracellular matrix (ECM) components [7]. Type I collagen, which constitutes about 80 to 90% of the skin, is mainly controlled by MMP-1 (type I collagenase) and MMP-3 (stromelysin-1), whereas MMP-2 (gelatinase-A) and MMP-9 (gelatinase-B) degrade gelatin; moreover MMP-2, MMP-7 (matrilysin-1), and MMP-12 (metalloelastase) degrade elastin [8]. Not only in response to UVB, UVA and blue light, the level of MMPs in the skin is highly up-regulated [3,4,9,10], therefore, the prevention of UVB-induced up-regulation of MMPs is one of the target pathways to inhibit wrinkle formation and prevent skin aging.

In living cells, ultraviolet (UV)-irradiation activates mitogen-activated protein kinase (MAPK) and regulates nuclear factor-kappa B (NF-κB) expression by generating reactive oxygen species (ROS) [3,4]. MAPK, is an enzyme family, consisting of three types: extracellular signal-regulated kinase (ERK), c-Jun N-terminal kinase (JNK), and p38 kinase, which are involved in cell proliferation, differentiation, apoptosis, and inflammation. The MAPK pathways also regulate the transcription factor activating protein 1 (AP-1), a heterodimer comprised of c-Fos and c-Jun, which, in turn, up-regulates MMPs in the skin [11,12].

Various synthetic materials have been developed as skin anti-aging agents and retinoid is the most popular among them. Retinoid is a synthetic analog of vitamin A consisting of retinoic acid and retinol (vitamin A) which promotes collagen synthesis in photoaged skin and inhibits the expression of MMPs [13]. Retinoic acid also promotes elastin and collagen synthesis [14]. However, retinoid is also reported to cause skin and liver toxicity and diseases, such as paronychia [14]. Owing to safety and lack of side effects, natural materials are being considered for the development of effective and safe anti-photoaging agents in the field of cosmetic research and development.

**Figure 1:**
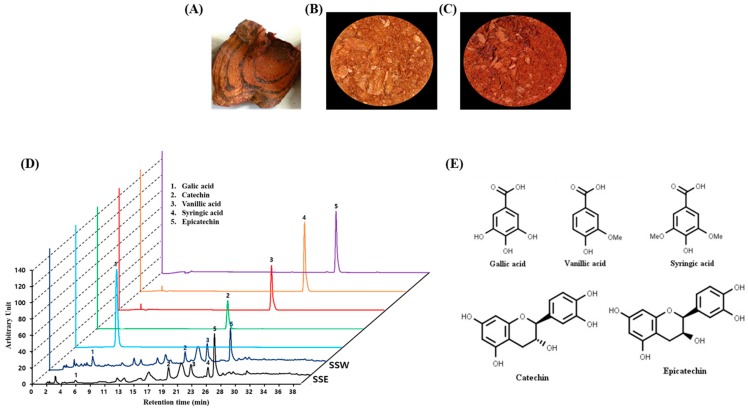
Classical feature of *Sapatholobus suberectus* (SS) and identification of ingredients. (**A**) Stem of *Sapatholobus suberectus*. (**B**,**C**) Powder of *Sapatholobus suberectus* stem’s aqueous (SSW) and ethanolic (SSE) extracts. (**D**) high-performance liquid chromatography (HPLC) profile of the stem of *Spatholobus suberectus*. (**E**) Molecular structure of the 5 compounds identified, gallic acid (1); catechin (2); vanillic acid (3); syringic acid (4); and epicatechin (5).

*Spatholobus suberectus* (SS) is a climbing shrub plant containing a red resin, belonging to the Leguminosae family, and mainly grows in China (Figure 1A). The stem of SS is known as “Gye-Hyeol-Deung” in Korea and “Ji Xue Teng” in China because it produces a red juice like chicken’s blood [15]. Traditionally, the stem of SS has been applied to treat inflammation-induced thrombosis and peripheral blood vessels [16]. Numerous scientific reports have revealed that SS has anti-hepatitis C virus activity [17], antiplatelet [18], anti-breast cancer [19], antioxidant [20], chondrogenesis stimulating [21], antiviral [22], and protective effects against cerebral ischemia [23]. It has also been reported that SS has the potential to regulate cartilage-related MMPs and TIMPs [21] and anti-inflammatory activity [23].

Based on previous reports, it was hypothesized that the aqueous and ethanolic extracts of *Spatholobus suberectus* stem (SSW and SSE, respectively) would play a pivotal role in human healthy skin homeostasis by mediating functionality and nutritional balance in body. However, there have been no reports regarding the potential dermatological application of the SS stem. SS extracts were considered in this study to evaluate its effects against photo-aging in human keratinocyte cultures and the underlying mechanism by which SS extract mitigates the appearance of wrinkles. Therefore, in this study, in order to assess what kinds of nutritional food ingredients are involved in the plant and how the nutrients can be applied for skin care for the better healthy life, we determined its effect on skin wrinkles and elasticity, as well as the mechanism to assess whether it has sufficient value as a natural material to suppress aging. This can contribute to the development of novel and useful cosmetic agents, supplements, and functional foods.

## 2. Materials and Methods

### 2.1. Plant Materials and Preparation of Plant Extracts

The stem of *Spatholobus suberectus* (SS) was purchased from a Chinese medicinal herb shop in Zhengzhou, China (Figure 1A). The sample was dried at 30 °C for two days and then pulverized into a fine powder. Ten grams of coarsely dried powder was extracted three times using 100% ethanol and distilled water under reflux for 3 h. The extract was decanted using filter paper (Whatman No. 1, Schleicher & Schuell, Keene, NH, USA). Then, the solvent was removed and dried using a rotary vacuum evaporator (Tokyo Rikakikai Co. Ltd., Tokyo, Japan) and finally pulverized after freeze-drying (Ilshin Biobase, Goyang, Korea) the aqueous and ethanolic extract of SS (SSW and SSE, respectively) (Figure 1B,C, respectively). Powdered extracts were dissolved in distilled water and stored at 4 °C until testing.

### 2.2. High-Performance Liquid Chromatography (HPLC) Analysis

The phytochemical characteristics of SSW and SSE were analyzed by high-performance liquid chromatography (HPLC) using standard compounds such as catechin, (−)-epigallocatechin gallate (EGCG, E4268, Sigma-Aldrich), epicatechin, gallic acid, syringic acid, and vanillic acid. The HPLC analysis was performed using a Shimadzu Prominence Auto Sampler (SIL-20A) HPLC system (Shimadzu, Kyoto, Japan), equipped with an SPD-M20A diode array detector (PDA) and LC solution 1.22 SP1 software. Before analysis, the samples were filtered through a 0.2 µm syringe filter (Pall Life Sciences, Ann Arbor, MI, USA). The reverse-phase chromatographic analysis was carried out using a Phenomenex C18 column (4.6 mm × 250 mm) packed with 5 µm diameter particles and maintained at 25 °C. A stepwise gradient of solvent A to B was used (A: 2% acetic acid and B: 50% acetonitrile (ACN) in 0.5% acetic acid) according to a previous report [24] with a slight modification. The flow rate was 1 mL/min, and 10 µL was injected.

### 2.3. Elastase Inhibition Assay and Combination Index

The elastase inhibition assay was performed according to a previously reported method with minor modifications [25]. Briefly, the reaction was carried out in a 0.1 M Tris-HCl buffer (pH 8.0) containing 0.78 mM N-Succinyl-(Ala)-3-*p*-nitroanilide (Sigma-Aldrich, St. Louis, MO, USA) as a substrate and 0.04 U elastase (Sigma-Aldrich, St. Louis, MO, USA). EGCG was used as a positive control. A predetermined concentration (10, 30, 100, or 300 μg/mL) of each sample was mixed thoroughly with 100 µL of the substrate solution and consequently 100 µL of the enzyme solution was added, and the absorbance was measured at 405 nm in a microplate reader (Wallac Victor3 1420 Multilabel Counter, Perkin Elmer, Waltham, MA, USA). The mode of interaction between syringic acid (SA), epicatechin (EP) and vanillic acid (VA) in inhibiting elastase was further analyzed by the CompuSyn program (ComboSyn Inc, Paramus, NJ, USA), which applies median effect equation methods. The combined drug effect is expressed as combination index (CI) versus fraction affected (Fa), with CI < 1 indicating synergism, CI = 1 indicating an additive effect, and CI > 1 indicating antagonism [26]. The CI values were calculated by the Chou–Talalay method based on the median-effect equation and the classic isobologram equation [26] using CompuSyn software (ComboSyn Inc, Paramus, NJ, USA).

### 2.4. Cell Culture, UVB-Irradiation and Cell Viability Assay

Human keratinocyte (HaCaT) cells were purchased from AddexBio Technologies (San Diego, CA, USA). Cells were grown in Dulbecco’s modified Eagle’s medium (DMEM) (Hyclone, Logan, UT, USA) supplemented with 10% fetal bovine serum (FBS) (Hyclone, Mordialloc, Victoria, Australia) and 1% penicillin-streptomycin (P/S) (Sigma-Aldrich, St. Louis, MO, USA) at 37 °C in a humidified atmosphere containing 5% CO_2_. Then, sub-confluent cells were treated with indicated concentration of SSW and SSE for 24 h. Subsequently, the cells were exposed to UVB at a dose of 40 mJ/cm^2^ using a UVB source (Bio-Link Crosslinker, Vilber Lourmat, Cedex, France) set at a spectral peak of 312 nm for 20 s. Rigel et al. reported healthy high school volunteers daily received the UVB irradiation by 8.01 mJ/cm^2^/day [27]. In this experiment, we used UVB radiation at 40 mJ/cm^2^, which is equivalent to approximately 5 days of sun exposure. After UVB irradiation, the cells were cultured in serum-free medium for 24 h. Cell viability was determined using the 3- (4,5-dimethylthiazol-2-yl) -2,5-diphenyltetrazolium bromide (MTT) colorimetric assay as described previously [28].

### 2.5. ROS Generation Assay

Intracellular ROS production in cells was detected using 2′,7′-Dichlorofluorescein diacetate (DCFH-DA) (Sigma-Aldrich, St. Louis, MO, USA) according to the methods described previously [26]. Cells were grown to 70–80% confluence then cultured (1 × 10^5^ cells/mL) with indicated concentration of SSW and SSE in 96-well black clear bottomed plates (Corning Inc., Corning, NY, USA) for 24 h and then exposed to UVB-irradiation (40 mJ/cm^2^), and further incubated for 24 h. Then, the cells were washed with phosphate-buffered saline (PBS) twice and treated with DCFH-DA (25 µM) for 30 min. Finally, fluorescence intensity was measured at excitation and emission wavelengths of 485 and 528 nm, respectively, by a fluorescence microplate reader (Victor3, PerkinElmer, Waltham, MA, USA).

### 2.6. RNA Extraction and Reverse Transcription-Polymerase Chain Reaction (RT-PCR)

HaCaT cells (1 × 10^5^ cells/mL) were cultured with indicated concentration of SSW and SSE (f.c. 3, 10, or 30 μg/mL) in 6-well plates for 24 h. Total RNA was isolated using TRIzol (Invitrogen, Carlsbad, CA, USA) following the manufacturer’s instructions. 2 µg of total RNA was used to prepare the complementary DNA (cDNA) using RT & GO Mastermix (MP Biomedicals, Seoul, Korea). A polymerase chain reaction (PCR) Thermal Cycler Dice TP600 (Takara Bio Inc., Otsu, Japan) was used to carry out RT-PCR using the various primer sequences (Table S1), according to the methods described earlier [28].

### 2.7. Preparation of Protein Lysates and Western Blotting

The lysates of HaCaT cells (2 × 10^5^ cells/mL) were prepared using radioimmunoprecipitation assay (RIPA) buffer with a phosphatase and protease inhibitor cocktail (Sigma-Aldrich, St. Louis, MO, USA) and the bicinchoninic acid (BCA) method was applied to quantify the protein content. A nuclear/cytosolic fractionation kit (Sigma-Aldrich, St. Louis, MO, USA) was used for the extraction of nuclear proteins. Aliquots of 30 μg of total proteins were used to carry out the Western blot analysis using various antibodies (Table S2) according to our previously described methods [28].

### 2.8. Statistical Analysis

The experimental data are presented as the mean ± standard deviation (SD). Enzyme, cell viability data were analyzed using a paired Student’s t-test. The statistical analysis of the rest of the data was carried out by one-way analysis of variance (ANOVA), followed by Dennett’s test using the SigmaPlot 12.5 (Systat Software Inc., San Jose, CA, USA). Differences were considered significant when ** *p* ≤ 0.05 or * *p* ≤ 0.01.

## 3. Results

### 3.1. HPLC Analysis of Stem of Spatholobus Suberectus (SS)

To gain insight into the phytochemicals present in SS stem extract, HPLC analysis was performed with standard phenolic and flavonoid compounds. Interestingly, the aqueous and ethanolic extracts of SS stem (SSW and SSE, respectively) (Figure 1B,C) demonstrated several peaks, with the retention times to be harmonized in the following standard compounds: gallic acid (6.380 min), catechin (20.074 min), vanillic acid (23.601 min), syringic acid (25.352 min) and epicatechin (26.809 min) (Figure 1D).

By using the peak areas of known concentrations of standards, the amounts of these polyphenols in *Spatholobus suberectus* stem extract were determined. As shown in Figure 1D, SSW contains gallic acid (6.90 µg/mL), syringic acid (27.08 µg/mL) and epicatechin (105.59 µg/mL) while SSE contains gallic acid (1.48 µg/mL), vanillic acid (21.07 µg/mL), syringic acid (28.23 µg/mL), catechin (1.86 µg/mL), and epicatechin (142.17 µg/mL) (Figure 1D). Both extracts commonly found to contain gallic acid, catechin, syringic acid and epicatechin.

### 3.2. Inhibition of Elastase Activity by SS Stem

**Figure 2:**
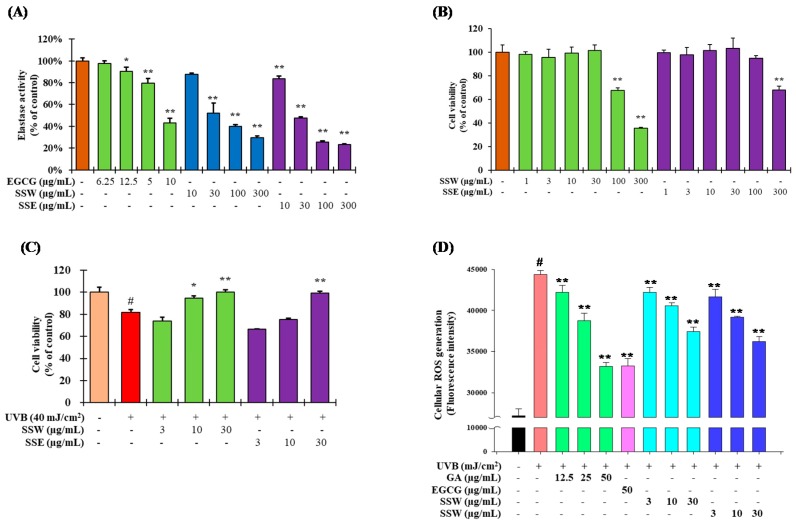
Comparison of anti-aging potential by SS. (**A**) Inhibitory effects of SS stem extracts on elastase activity. The results are denoted as the mean ± standard deviation (SD) from triplicate experiments (** *p* ≤ 0.01, * *p* ≤ 0.05 compared with the control group). (**B**) Cytotoxicity of SS stem extracts in HaCaT cells. Cells (1 × 10^5^ cells/mL) were seeded in a 96-well plate and treated with aqueous and ethanolic extracts of SS stem (SSW and SSE) at 1, 3, 10, 30, 100, and 300 µg/mL. The cell viability was determined using 3- (4,5-dimethylthiazol-2-yl) -2,5-diphenyltetrazolium bromide (MTT) assay. (**C**) Phototoxicity of SS stem extracts in HaCaT cells. Cells (1 × 10^5^ cells/mL) were treated with SSW and SSE at 3, 10, or 30 µg/mL. After 24 h, UVB (40 mJ/cm^2^) was irradiated and the cells were cultured in serum-free medium for 24 h. The cell viability was measured using the MTT assay. The data are denoted as the mean ± SD from triplicate results. (# *p* ≤ 0.01, compared with the non treated control (NT); ** *p* ≤ 0.01, * *p* ≤ 0.05 compared with the UV control group). (**D**) Reactive oxygen species (ROS) generation activity was measured using HaCaT cells according to the method described in materials and methods. EGCG: (−)-epigallocatechin gallate; GA: gallic acid.

The elastase inhibition assay revealed both SSW and SSE were suppressed the elastase activity in a concentration-dependent manner (Figure 2A). At 100 µg/mL, SSE showed 79% inhibition of elastase activity whereas EGCG (used as positive control) and SSW had similar inhibitory activity as 59%. The IC_50_ values of the elastase inhibition activity were in the order of SSE > SSW > EGCG. These results suggest that SSW and SSE were found to suppress the breakdown of elastin, which in combination with collagen contributes to skin wrinkles.

### 3.3. The Effet of SS on Cell Viability of Human Keratinocytes (HaCaT) Cells

Cell viability was determined using the MTT assay in HaCaT cells (Figure 2B). The cell viability of approximately 80% was considered to be non-toxic. SSW did not show any toxicity up to a concentration of 30 µg/mL, but the survival rate was decreased to 68% at a concentration of 100 µg/mL. On the other hand, SSE showed no cell toxicity up to 100 µg/mL, but the survival rate was approximately 68% at a concentration of 300 µg/mL. Therefore, 30 µg/mL was considered the highest concentration for the subsequent experiments for both samples.

To determine the UVB intensity to be used in the analysis, the survival rate of the cells was examined by irradiating them with UVB. Cells were irradiated with UVB at 0, 30, 40, and 50 mJ/cm^2^ for cytotoxicity according to UVB irradiation intensity. The survival rate of the HaCaT cells was about 86% at 30 mJ/cm^2^ and 75% at 40 mJ/cm^2^ (Figure S1). As shown in Figure 2C, UVB irradiation significantly reduced the cell viability, while pretreatment of both SSW and SSE alleviated the harmful effect of radiation to the cells. Upon UVB irradiation, the cell viability was reduced by 18% whereas pretreatment with both SSW and SSE restored the cell survivability. Surprisingly, at a concentration of 30 µg/mL of SSW and SSE, both extracts completely abolished the effects of UV-induced cell damage (Figure 2C).

### 3.4. Suppression of UVB-Stimulated Reactive Oxygen Species (ROS) Generation by SS

The goal was to investigate whether SSW and SSE suppress the UVB-stimulated ROS production. As described in Figure 2D, UVB irradiation significantly enhanced ROS generation compared with the non-irradiated cells. Pretreatment of SSW and SSE significantly lessened ROS generation compared with the UV-irradiated control. At a concentration of 30 µg/mL, both SSW and SSE decreased ROS production about 11% and 18%, respectively. On the other hand, both gallic acid and EGCG suppressed almost 50% of UVB stimulated ROS generation at 50 µg/mL.

### 3.5. Regulation of UVB-Induced Matrix Metalloproteinases (MMPs) Expression by SS

**Figure 3:**
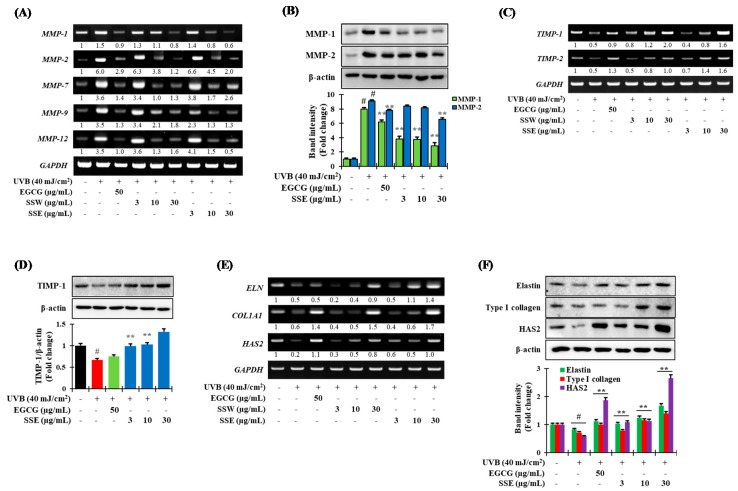
Effects of aging-related biomarkers by SSE. The effects of SSE on the regulation of (**A**) the mRNA expression and (**B**) the protein level of MMPs; (**C**) the mRNA expression and (**D**) the protein level of tissue inhibitors of matrix metalloproteinases (TIMPs); (**E**) the mRNA expression and (**F**) the protein level of elastin (ELN), type I collagen (COL1A1) and hyaluronan synthase 2 (HAS2) induced by UVB (40 mJ/cm^2^) in HaCaT cells. Cells were cultured for 24 h, and then with the indicated concentration of SSW and SSE for further 12 h. Then the cells were irradiated with 40 mJ/cm^2^ UVB and cultured for additional 12 h. Reverse transcription-polymerase chain reaction (RT-PCR) and western blot was carried out according to the methods described in materials and methods. The data are denoted as the mean ± SD from triplicate results. (*# p* ≤ 0.01, compared with the NT; ** *p* ≤ 0.05, compared with the ultraviolet (UV) control group). EGCG: (−)-epigallocatechin gallate used as a positive control.

In order to confirm the capability of SSE to regulate UVB-stimulated MMPs expression, HaCaT cells were pretreated by SSE followed by subjected to expose in UVB (40 mJ/cm^2^), and the expression of MMPs was determined by RT-PCR and immunoblotting assay. As described in Figure 3A,B, pretreatment of SSE significantly abolished the UVB-stimulated MMPs mRNA level in a dose-dependent fashion. Interestingly, the suppressive effect of SSE on MMP-1 and -2 expression was higher than that of EGCG used as a positive control (Figure 3B). Furthermore, the results show that pretreatment with both SSW and SSE was significantly reduced in UV-induced MMP-12 transcriptional levels in a concentration-dependent fashion (Figure 3A).

Furthermore, to confirm whether SSE could regulate the TIMPs expression in UVB-stimulated HaCaT cells, both RT-PCR and western blotting assay were carried out. As described in Figure 3C,D, UVB treatment downregulated the TIMP expression compared to non-treated cells, while SSE pretreatment further halted this action.

### 3.6. Effects of SSE on the Expression of COL1A1, ELN and HAS2

Skin wrinkles and elasticity are mainly influenced by collagen, elastin, and hyaluronic acid. Therefore, to examine whether the SS stem extracts had the ability to regulate these biomarkers, the transcriptional and translational expression of elastin (ELN), type I collagen (COL1A1) and hyaluronan synthase 2 (HAS2) was confirmed by RT-PCR and immunoblotting, respectively (Figure 3E,F). The results show that UVB treatment drastically downregulated the transcriptional and translational level of elastin, type I collagen, and hyaluronan synthase 2, while pretreatment of SSE fixed their expression.

### 3.7. Downregulation of NF-κB and AP-1 by SSE

**Figure 4:**
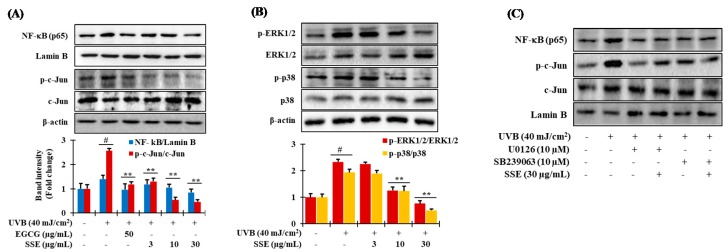
Modulation of mitogen-activated protein kinase (MAPK)/nuclear factor-kappa B (NF-κB) and AP-1 signaling by SSE in UVB-stimulated HaCaT cells. (**A**) The effect of SSE on the phosphorylation of NF-κB, p-c-Jun and c-Jun modulated by UVB. Cells were cultured for 24 h, and then SSE were added for 12 h. After that, UVB was irradiated at 40 mJ/cm^2^, and the protein expression was confirmed by western blot as described in materials and methods. (**B**) The effects of SSE on the phosphorylation of mitogen-activated protein (MAP) kinase activated by UVB. A 12 h pretreated cells by SSE, UVB was irradiated at 40 mJ/cm^2^, and the protein was extracted after 30 min. Protein expression was confirmed by western blot. (**C**) Specific extracellular signal–regulated kinases (ERK) and p38 inhibitors (U0126 and SB239063, respectively) manifest the effects of SSE on the regulation of NF-κB and AP-1 expression in UVB-irradiated HaCaT cells. Cell were treated according to the above-mentioned protocol and protein expression was evaluated by immunoblotting. (# *p* ≤ 0.05, compared with the NT; ** *p* ≤ 0.05, compared with the ultraviolet (UV) control group). EGCG: (−)-epigallocatechin gallate.

It is well known that the transcriptional levels of various MMPs are strongly modulated by NF-κB and AP-1. They also play significant roles in maintaining the ECM composition as well as in cytokine expression [29]. Because SSE stifled the expression of MMPs, we next examined whether SSE was able to regulate the transcription factors of NF-κB and AP-1 which is responsible for MMPs expression. To estimate the modifications of the expressions of NF-κB and AP-1 transcription factors induced by SSE in UVB-treated HaCaT cells, we turned to immunoblotting. The protein levels of NF-κB family members, p65, were significantly raised by UVB but lessened by SSE in a concentration dependent fashion (Figure 4A, upper layer). Likewise, SSE also considerably reduced the protein levels of p-c-Jun, which are the components of AP-1 (Figure 4A, lower layer).

### 3.8. Effects of SSE on the Phosphorylation of Mitogen-Activated Protein Kinase (MAPK) Proteins

Next, we investigated the pathway through which SSE exhibits its anti-photoaging effects. Generally, UVB induced augmented ROS production leads to the activation of MAPK proteins including extracellular signal–regulated kinases (ERK), p38 mitogen-activated protein kinases (p38) and c-Jun N-terminal kinases (JNK). MAPK induces NF-κB and AP-1 which consequently boosts the expression of MMPs, lead to decrease of collagen and other ECM in aged skin [30]. To investigate the effects of SSE on UVB-induced photoaging, the phosphorylation of MAPKs was assessed. UVB-irradiation significantly amplified the phosphorylation of ERK, and p38, compared with non-irradiated cells and peak at 30 min after UVB exposure (Figure S2), while the phosphorylation of JNK was minimal. Our data are consistent with studies by Chouinard et al. where it was shown that UVB failed to activate JNK, albeit the induction was more pronounced for the phosphorylation of ERK1/2 and p38 [31]. Treatment with SSE at 30 µg/mL provided the most inhibition on phosphorylated ERK1/2 and p38 (Figure 4B) whereas the inhibition effects of SSE on the phosphorylation of JNK was absent. Thus, to validate whether SSE-modulated downregulation of NF-κB and AP-1 is associated with the MAPK signaling cascade, cells were treated with specific p38 and ERK1/2 inhibitors, such as SB239063 and U0126, respectively, before being treated with SSE. Both p38 and ERK1/2 inhibition, along with SSE, exhibited the potential to attenuate the protein expression of NF-κB and AP-1 (Figure 4C). These data show that the suppression of UVB-stimulated ERK and p38 phosphorylation by SSE are required in the attenuation of NF-κB and AP-1 in HaCaT cells.

### 3.9. Combination Studies of the Major Components of SSE on Elastase Inhibition Activity

**Figure 5:**
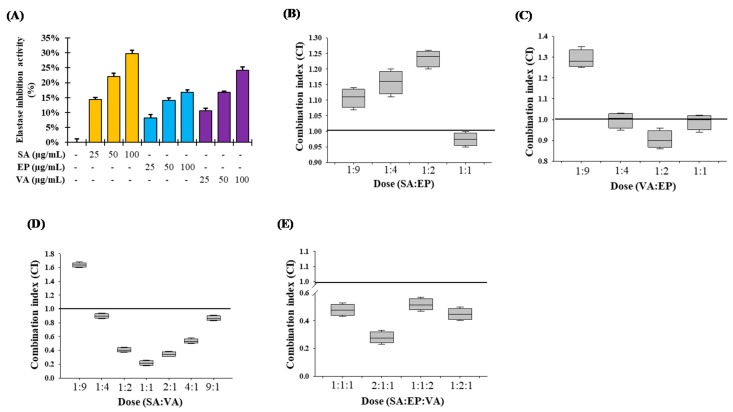
Individual and combination effects of elastase inhibition by the major identified compounds of SSE. (**A**) Elastase inhibition activity of the major identified compounds of SSE. The results are denoted as the mean ± SD from triplicate experiments. (**B**–**E**) Combination effects of elastase inhibition by the major identified compounds of SSE. Combination index (CI) values of <1, =1, and >1 indicate synergism, additive effect, and antagonism, respectively. CI values shown are mean ± SE with a minimum of three experiments. SA: syringic acid; EP: epicatechin and VA: vanillic acid. The raw compuSyn report are given in supplementary data.

The major compounds of SSE identified were syringic acid (SA), epicatechin (EP) and vanillic acid (VA), and all of them showed dose-dependent moderate elastase inhibitory activity (Figure 5A). These results suggest that SA, EP and VA could inhibit elastase activity by different pathways and that combination of these agents might result in boosted inhibitory effects. Thus, we compared the inhibitory potencies of SA, EP and VA and their combinations (SA and EP; VA and EP, SA and VA at 1:9, 1:4, 1:2 and 1:1 ratio as well as SA, VA and EP at 1:1:1, 2:1:1, 1:2:1 and 1:1:2 ratio) on elastase inhibition activity. Combinations of equimolar doses of SA and EP showed nearly additive effects (CI = 0.97) while other all combination doses showed slight antagonism (Figure 5B). As described in Figure 5C, VA and EP combinations also showed nearly additive effects in all combinations (CI = 0.99, 0.91 and 0.99 at 1:4, 1:2 and 1:1 ratio, respectively) except 1:9 (VA:EP) ratio. These results suggested that the lower concentration of EP showed additive effects by combining with SA and/or VA. Moreover, combination of SA and VA at 1:2, 1:1, and 2:1 ratio showed very good synergistic effects (CI = 0.41, 0.22 and 0.37, respectively) on elastase inhibition (Figure 5D). Furthermore, strong synergism was observed at 2:1:1 molar ratio of SA, VA and EP (CI = 0.28), while other combinations of these three compounds also gave a moderate synergism effect (Figure 5E). Our experiments demonstrated that only lower concentrations of EP with SA and/or VA had additive effects, while combination of SA, VA and EP at lower concentrations was potently synergistic.

## 4. Discussion

Epidemiological studies revealed that overexposure of UV irradiation significantly increases the magnitude of patients with skin damage. Chronical exposure of UV irradiation to skin causes oxidative stress, inflammation, ROS-mediated DNA damage, and disorder of cellular signaling pathways, resulting in accelerated skin photo-aging [32,33]. Botanicals possessing antioxidant, anti-inflammatory and immunomodulatory properties are promising to be exploited as therapeutic agents for a variety of skin disorders, including photo-aging [34,35]. *Spatholobus suberectus* (SS) stem was found to contain various polyphenolics, such as gallic acid, vanillic acid (VA), epicatechin (EP), and syringic acid (SA) (Figure 1D). Among them, gallic acid has been reported to be effective for anti-aging in human fibroblasts through wound healing [36], and syringic acid has been reported to be effective for elastase inhibition [37]. Indeed, various photochemopreventive agents derived from dietary origin have shown the potential efficacy on skin delivery through oral systemic administration, an emerging concept referred to as ‘nutritional’ or ‘systemic photoprotection’. The systemic administration of an apocarotenoid bixin, an FDA-approved natural food colorant from the seeds of the achiote tree (*Bixa orellana*), protect the solar UV-induced skin damage in SKH-1 mice through the activation of cutaneous Nrf-2 signaling [38]. In this study, the aqueous and ethanolic extracts of SS stem’s (SSW and SSE, respectively) significantly inhibited elastase activity (Figure 2A), possibly due to the presence of SA, VA and EP, which may cause synergism on elastase inhibition (Figure 5). Furthermore, HaCaT cells, which are human epidermal keratinocytes, were considered to prove the underlining mechanism of the anti-photoaging activity of SSE. Before experimentation, the toxicity of the extracts to the cells was confirmed. Both SSW and SSE were found to be non-toxic in HaCaT cells up to a concentration of 30 µg/mL (Figure 2B). Therefore, the following concentrations were used in subsequent experiments.

MMPs play an important role in the physiological mechanisms of skin photo-aging. UV irradiation alters the connective tissues of the skin by up-regulating the expression of MMPs, which degrade collagen and other ECM proteins. SSE has been shown to suppress UVB-induced upregulation of MMP-1 and -2 (Figure 3A,B). MMP-1 is a major collagenase that causes collagen degradation in skin severely damaged by UV. MMP-12 is the most important elastin-degrading enzyme and is involved with elastin and many other substrates, such as ECM collagen [39]. The tissue inhibitors of metalloproteinases or TIMPs suppress MMP activity critical for extracellular matrix turnover associated with both physiologic and pathologic tissue remodeling. TIMP concentrations generally far exceed the concentration of MMPs in tissue and extracellular fluids, thereby limiting their proteolytic activity to focal pericellular sites [40]. Treatment of SSE significantly boosted the TIMP-1 and -2 expression than that of UVB-stimulated cells (Figure 3C,D)

Notably, collagen is the principal protein that connects skin tissue, and the decomposition of collagen produces wrinkles in the skin. COL1A1 is one of the genes that constitute type I collagen and decreases upon exposure of UV [5,6]. Elastin is an extracellular matrix protein that binds to collagen and provides elasticity to the skin or elastic tissue. It is reduced by the activation of elastase upon UVB exposure [41]. When collagen and elastin are combined, hyaluronic acid plays a supporting role. Hyaluronan synthase 2 (HAS2) is an enzyme that synthesizes hyaluronic acid and plays a role in supporting the structure of the skin. In addition, HAS2 is abolished upon UVB skin irradiation [42]. SSE treatment significantly hindered the UVB-induced downregulation of type I collagen, elastin and HAS2 expression in concentration dependent manner (Figure 3E,F), which also supports the result that treatment with SSE increases cell proliferation.

**Figure 6:**
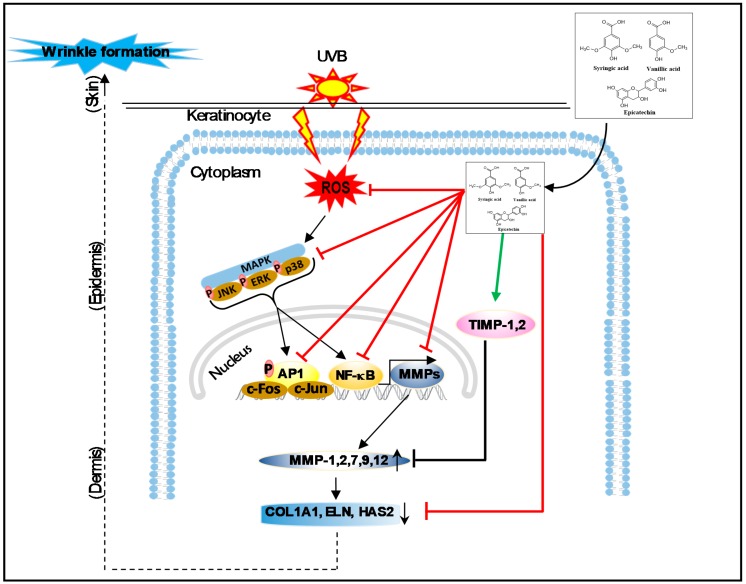
A proposed mechanism of SSE against UVB-induced photo-aging.

UV irradiation stimulates cell surface growth factor receptors, cytokines, and MAPKs, which in turn regulate AP-1. Increased AP-1 activity down-regulates type I pro-collagen and up-regulates MMP-1. MMP-1 gene expression is regulated by c-Jun and c-Fos, which are components of the AP-1 heterodimer complex [33]. Moreover, it has been well established that a transcription factor, nuclear factor κB (NF-κB), is activated in skin keratinocytes by UV irradiation and leads to the augmentation of the levels of MMP-1, thus suppression of NF-κB pathway would freeze the UVB-induced skin photoaging process [43]. Interestingly, SSE treatment significantly suppressed UV-induced c-Jun expression (Figure 4B), which may inhibit AP-1 activity as well as NF-κB signaling cascade resulting in suppression of MMPs expression. Our results are also in accordance with the previous studies [44], in which the active parthenolide, a sesquiterpene lactone compound of *Tanacetum parthenium*, completely blocked the NF-κB signaling pathway and hindered the UVB-mediated cutaneous alteration in both cultured cell and animal model. Furthermore, *Terminalia catappa* water extract protected skin from photodamage by inhibiting the MAPK/AP-1/MMP pathway [45]. Cumulating evidence has shown that exposure of the skin to UV induces ROS generation triggers MAPKs (ERK, JNK, and p38) phosphorylation, NF-κB, AP-1 activation, and regulates the expression of genes and proteins such as MMPs, leading to collagen degradation, resulting in photodamage and photo-carcinogenesis [41,43]. In the present study, UVB stimulation upregulated the phosphorylation of MAPKs, AP-1, NF-κB and MMPs, while SSE significantly suppressed these effects (Figure 4). Pharmacological inhibition of these signaling cascades abolished SSE-induced nuclear accumulation of AP-1, and NF-κB (Figure 4C). This finding suggests that SSE activity is dependent on this signaling transduction. The possible mechanisms of action of the *Spatholobus suberectus* stem extract against skin photoaging are summarized in Figure 6.

## 5. Conclusions

In this study, SS stem extract inhibited the ROS production by UV as well as repressing UV-activated MAP kinase, and ultimately enhanced the expression of COL1A1, ELN and HAS2. The active components, which were identified by HPLC as gallic acid, catechin, vanillic acid, syringic acid, and epicatechin, not only affect protection from ROS and cellular damage, but also inhibition of MMPs, activation of COL1A1, ELN, and HAS2, eventually blocking the phosphorylation of MAPKs and its downstream transcription factor such as AP-1, and NF-κB. These results collectively suggest that *Spatholobus suberectus* stem extract seems to be valuable as a natural biomaterial that can inhibit UVB-stimulated photo-aging.

## Supplementary Materials

The following are available online at https://www.mdpi.com/2072-6643/11/6/1341/s1, Figure S1: Cell viability UVB-irradiation. HaCaT cells (1 × 105 cells/mL) were seeded in a 96-well plate and treated with UVB-irradiation at 20, 30, 40 and 50 mJ/cm^2^. The cell viability was determined using the MTT assay, Figure S2: The effects of UVB-irradiation on the phosphorylation of MAP kinase in HaCaT cells. HaCaT cells (1 × 105 cells/mL) were seeded in a 6-well plate and treated with UVBirradiation at 40 mJ/cm^2^ and the protein was extracted after 15, 30, 60 and 120 min Protein expression was confirmed by western blot, Table S1: List of the primer sets used in this study, Table S2: List of antibodies used in this study.
