# Supplementary material for: Attenuation of UVB-Induced Photo-Aging by Polyphenolic-Rich Spatholobus Suberectus Stem Extract Via Modulation of MAPK/AP-1/MMPs Signaling in Human Keratinocytes
Source: Nutrients. 2019 Jun 14;11(6):1341. doi: 10.3390/nu11061341 (PMC6627339; doi:10.3390/nu11061341)
Supplement: Supplementary file 1 [file nutrients-11-01341-s001.pdf]

SUPPLEMENTARY DATA SHEETS

Attenuation of UVB-induced Photoaging by polyphenolic rich Spatholobus suberectus stem extract via modulation of MAPK/AP-1/MMPs signaling in human keratinocytes

Kyoo-Ri Kwon[1†,$], Md Badrul Alam[1,2,$], Peijun Zhao[1], Ji-Hyun Park[1], Tae-Ho Kim[3], and Sang-Han Lee[1,2*]

[1]Department of Food Science & Biotechnology, Kyungpook National University, Daegu 41566, Republic of Korea

[2]Food and Bio-Industry Research Institute, Inner Beauty/Anti-ageing Center, Kyungpook National University, Daegu 41566, Republic of Korea

[3]Biomedical Research Institute, Kyungpook National University Hospital, Daegu 41944, Republic of Korea

$Authors are contributed equally

Correspondence to: Dr. Sang-Han Lee,

E-mail: sang@knu.ac.kr

[†]Present address: Biomedical Research Institute, Kyungpook National University Hospital, Daegu 41944, Republic of Korea

Table S1: List of the primer sets used in this study

Gene name		*Sequences*
MMP-1	*forward*	*CCTAGCTACACCTTCAGTGG*
	reverse	*GCCCAGTACTTATTCCCTTT*
MMP-2	*forward*	*GATACCCCTTTGACGGTAAGGA*
	reverse	*CCTTCTCCCAAGGTCCATAGC*
MMP-7	*forward*	*GTATGGGACATTCCTCTGATCC*
	reverse	*CCAATGAATGAATGAATGGATG*
MMP-9	*forward*	*TGTACCGCTATGGTTACACTCG*
	reverse	*GGCAGGGACAGTTGCTTCT*
MMP-12	*forward*	*CATGAACCGTGAGGATGTTGA*
	reverse	*GCATGGGCTAGGATTCCACC*
TIMP-1	*forward*	*TGGTGGGTGGATGAGTAATG*
	reverse	*CGCTGGTATAAGGTGGTCTG*
TIMP-2	*forward*	*ATGCACATCACCCTCTGTGA*
	reverse	*CTCTGTGACCCAGTCCATCC*
COL1A1	*forward*	*GAGGGCCAAGACGAAGACATC*
	reverse	*CAGATCACGTCATCGCACAAC*
ELN	*forward*	*CAAGGCTGGTTACCCAACAG*
	reverse	*CACCTGGGACAACTGGAATC*
HAS2	*forward*	*GAGCACCAAGGTTCTGCTTC*
	reverse	*CTCTCCATACGGCGAGAGTC*
Gapdh	*Forward*	*TTGTGATGGGTGTGAACCAC*
	reverse	*ACACATTGGGGGTAGGAACA*

Table S2: List of antibodies used in this study

Name	Catalog number	Company	Antigen	Host
Anti-MMP1	sc-21731	Santa Cruz Biotechnology, Inc.	MMP-1	Mouse
Anti-MMP-2	BS90870	Bioworld Technology, Inc.	MMP-2	Rabbit
Anti-TIMP-1	BS1697	Bioworld Technology, Inc.	TIMP-1	Rabbit
Anti-type I procollagen	sc-376350	Santa Cruz Biotechnology, Inc.	COL1A1	Mouse
Anti-ELN	BS7427	Bioworld Technology, Inc.	ELN (Elastin)	Rabbit
Anti-HAS2	sc-365263	Santa Cruz Biotechnology, Inc.	HAS2	Mouse
Anti-p-p38	sc-166182	Santa Cruz Biotechnology, Inc.	p38	Mouse
Anti-p38	BS3567	Bioworld Technology, Inc.	p38	Rabbit
Anti-p-ERK1/2	sc-7383	Santa Cruz Biotechnology, Inc.	ERK	Mouse
Anti-ERK1/2	BS 6472	Bioworld Technology, Inc.	ERK	Rabbit
Anti-p-JNK	BS 4322	Bioworld Technology, Inc.	JNK	Rabbit
Anti-JNK	sc-7345	Santa Cruz Biotechnology, Inc.	JNK	Mouse
Anti-NF- B (p65)	BS1254	Bioworld Technology, Inc.	NF- B (p65)	Rabbit
Anti-p-c-Jun	BS4050	Bioworld Technology, Inc.	c-Jun (Y-170)	Rabbit
Anti-c-jun	MB0004	Bioworld Technology, Inc.	c-Jun	Rabbit
Anti Lamin B	Sc-6217	Santa Cruz Biotechnology, Inc.	Lamin B	Goat
Anti- actin	Sc-47778	Santa Cruz Biotechnology, Inc.	-actin	Mouse

57

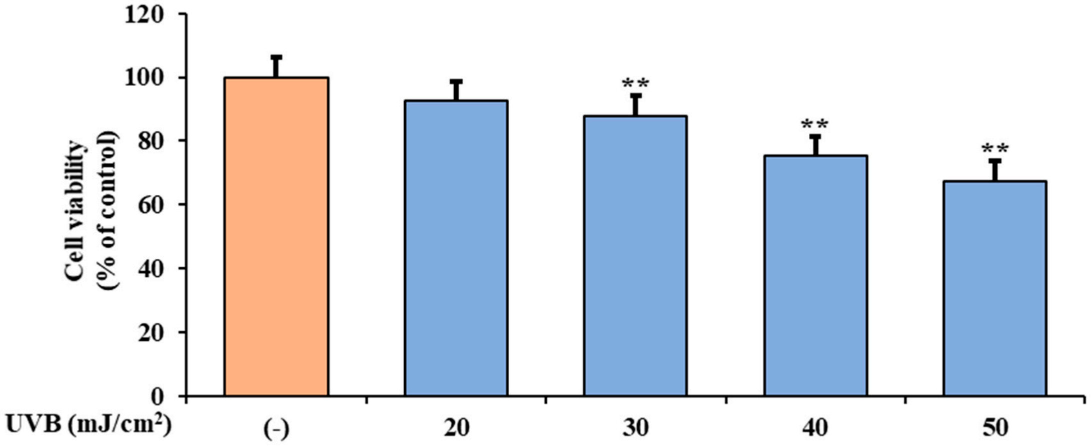

Figure S1. Cell viability UVB-irradiation. HaCaT cells (1 × 10^5 cells/mL) were seeded in a 96-well plate and treated with UVB-irradiation at 20,30,40 and 50 mJ/cm^2. The cell viability was determined using the MTT assay.

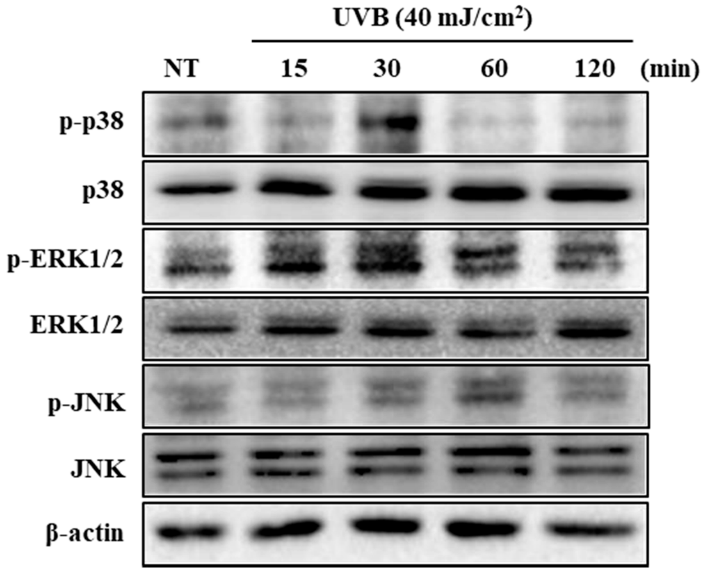

Figure S2. The effects of UVB-irradiation on the phosphorylation of MAP kinase in HaCaT cells. HaCaT cells (1 × 10^5 cells/mL) were seeded in a 6-well plate and treated with UVBirradiation at 40 mJ/cm^2 and the protein was extracted after 15, 30, 60 and 120 min Protein expression was confirmed by western blot.

CompuSyn Report

Experiment Name: combination effect

Date:

File Name: E:\koori\combination report.cse

Description

Drug: syringic acid (SA) [ug/mL]

Drug: Epicatechin (EP) [ug/mL]

Drug: Vanilic acid (VA) [ug/mL]

Drug Combo: combination 1 (SAEP) (SA+EP)

Drug Combo: combination 2 (EPVA) (EP+VA)

Drug Combo: combination 3 (SAVA) (SA+VA)

Drug Combo: combination 4 (SEV) (SA+EP+VA)

Data for Drug: SA [ug/mL] **Dose Effect**

0.25	0.18
0.5	0.26
1.0	0.31

3 data points entered.

X-int: 0.64112

Y-int: -0.3312 +/- 0.03634

m: 0.51665 +/- 0.09351

Dm: 4.37645 **r:** 0.98401

Data for Drug: EP [ug/mL] **Dose Effect**

0.25	0.2
0.5	0.24
1.0	0.3

3 data points entered.

X-int: 0.95979

Y-int: -0.3732 +/- 0.01162

m: 0.38880 +/- 0.02989

Dm: 9.11578 **r:** 0.99706

Data for Drug: VA [ug/mL] **Dose Effect**

0.25	0.15
0.5	0.2
**Dose**	**Effect**
1.0	0.28

3 data points entered.

X-int: 0.73153
Y-int: -0.4169 +/- 0.01514
m: 0.56997 +/- 0.03895
Dm: 5.38922 **r:**
0.99767

97
---
98 Data for Non-Constant Combo: SAEP (SA+EP)

Dose SA Dose EP Effect

1.0 9.0 0.51 1.0
4.0 0.45 1.0
2.0 0.39
1.0 1.0 0.38 99
4 data points entered.

100
---
101 Data for Non-Constant Combo: EPVA (EP+VA)

Dose EP Dose VA Effect

9.0 1.0 0.49 4.0
1.0 0.45 2.0
1.0 0.41
1.0 1.0 0.36 102
4 data points entered.

103
---
104 Data for Non-Constant Combo: SAVA (SA+VA)

Dose SA Dose VA Effect

1.0 9.0 0.52
1.0 4.0 0.5
1.0 2.0 0.55 1.0
1.0 0.58 2.0
1.0 0.57 4.0
1.0 0.59
9.0 1.0 0.62 105
7 data points entered.

106
---
107 Data for Non-Constant Combo: SEV (SA+EP+VA)

Dose SA Dose EP Dose VA Effect

1.0 1.0 1.0 0.51 2.0
1.0 1.0 0.62 1.0
1.0 2.0 0.54
1.0 2.0 1.0 0.52 108 4
data points entered.

Dose-Effect Curve for Drug Combos

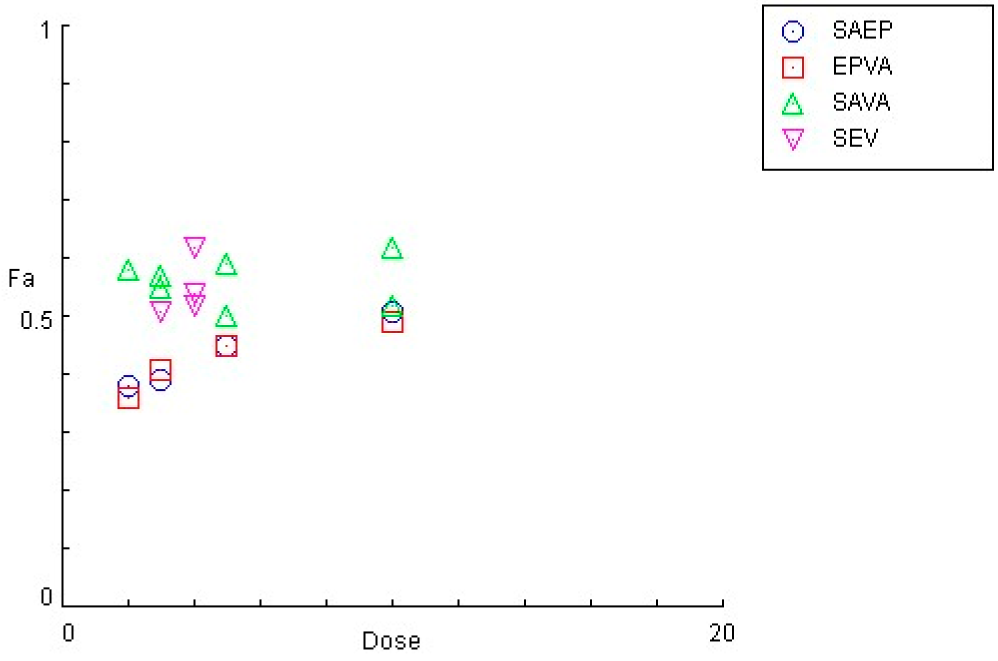

CI Data for Non-Constant Combo: SAEP (SA+EP)

**Dose SA**	**Dose EP**	**Effect**	**CI**
1.0	9.0	0.51	1.10223
1.0	4.0	0.45	1.07216
1.0	2.0	0.39	1.23635
1.0	1.0	0.38	0.97577

CI Data for Non-Constant Combo: EPVA (EP+VA)

**Dose EP**	**Dose VA**	**Effect**	**CI**
9.0	1.0	0.49	1.29334
4.0	1.0	0.45	0.99908
2.0	1.0	0.41	0.91088
1.0	1.0	0.36	0.99102

CI Data for Non-Constant Combo: SAVA (SA+VA)

**Dose SA**	**Dose VA**	**Effect**	**CI**
1.0	9.0	0.52	1.64690
1.0	4.0	0.5	0.97072 1.0
2.0	0.55	0.41593 1.0	
1.0	0.58	0.22766 2.0	
1.0	0.57	0.37801 4.0	
1.0	0.59	0.54983	
9.0	1.0	0.62	0.87588

CI Data for Non-Constant Combo: SEV (SA+EP+VA)

Dose SA Dose EP Dose VA Effect CI

1.0 1.0 1.0 0.51 0.48342 2.0

1.0 1.0 0.62 0.28692 1.0

1.0 2.0 0.54 0.52027

1.0 2.0 1.0 0.52 0.53552

Combination Index Plot

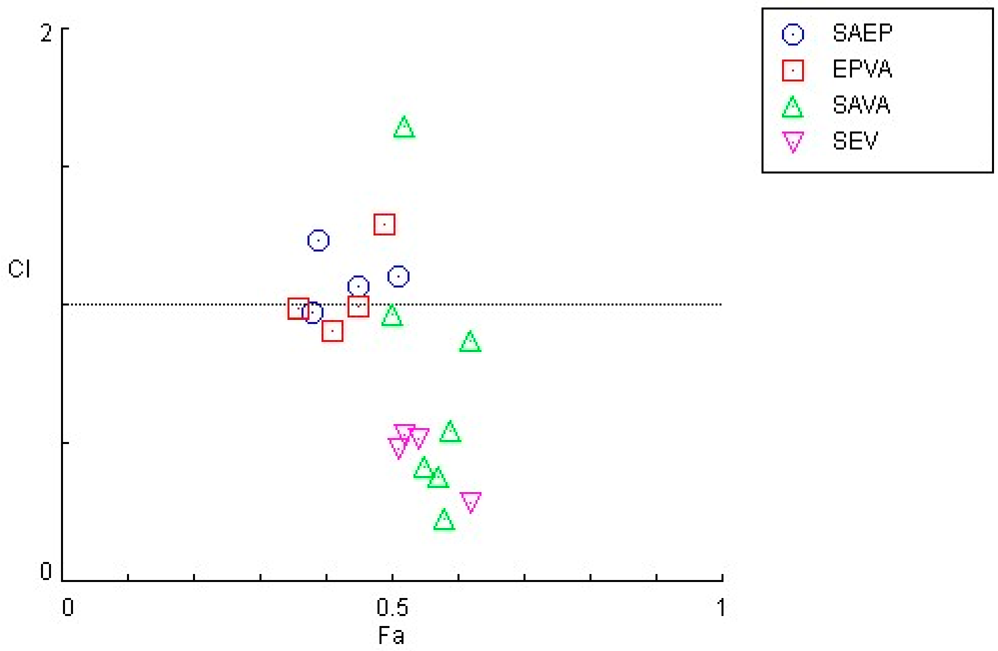

DRI Data for Non-Constant Combo: SAEP (SA+EP) **Fa Dose SA**

Dose EP DRI SA DRI EP

0.51 4.72880 10.1037 4.72880 1.12263 0.45

2.96783 5.44057 2.96783 1.36014 0.39

1.84125 2.88500 1.84125 1.44250 0.38

1.69671 2.58802 1.69671 2.58802

DRI Data for Non-Constant Combo: EPVA (EP+VA) **Fa Dose EP**

Dose VA DRI EP DRI VA

0.49 8.22447 5.02392 0.91383 5.02392 0.45

5.44057 3.78984 1.36014 3.78984 0.41

3.57473 2.84575 1.78737 2.84575 0.36

2.07544 1.96389 2.07544 1.96389

DRI Data for Non-Constant Combo: SAVA (SA+VA) **Fa Dose SA**

Dose VA DRI SA DRI VA

0.52 5.10982 6.20177 5.10982 0.68909

Fa Dose SA Dose VA DRI SA DRI VA

0.5 4.37645 5.38922 4.37645 1.34730

0.55 6.45365 7.66355 6.45365 3.83178 0.58

8.17419 9.49442 8.17419 9.49442 0.57

7.55172 8.83663 3.77586 8.83663 0.59

8.85260 10.2060 2.21315 10.2060

0.62 11.2885 12.7217 1.25428 12.7217

DRI Data for Non-Constant Combo: SEV (SA+EP+VA)

Fa Dose SA Dose EP Dose VA DRI SA DRI EP DRI VA

0.51 4.72880 10.1037 5.78107 4.72880 10.1037 5.78107
0.62 11.2885 32.1085 12.7217 5.64424 32.1085
12.7217 0.54 5.96906 13.7688 7.14005 5.96906
13.7688 3.57003

0.52 5.10982 11.1996 6.20177 5.10982 5.59979 6.20177

Summary Table

Experiment Name: combination effect

Date:

File Name: E:\koori\combination report.cse

Description

Drug: syringic acid (SA) [ug/mL]

Drug: Epicatechin (EP) [ug/mL]

Drug: Vanilic acid (VA) [ug/mL]

Drug Combo: combination 1 (SAEP) (SA+EP)

Drug Combo: combination 2 (EPVA) (EP+VA)

Drug Combo: combination 3 (SAVA) (SA+VA)

Drug Combo: combination 4 (SEV) (SA+EP+VA)

**Drug/Combo**	**Dm**	**m**	**r**
SA	4.37645	0.51665	0.98401
EP	9.11578	0.38880	0.99706
VA	5.38922	0.56997	0.99767

CI values at:

Combo ED50 ED75 ED90 ED95

Data for Fa = 0.5

Drug/Combo CI value Dose SA Dose EP Dose VA

SA		4.37645		
EP			9.11578	
VA				5.38922

Data for Fa = 0.75

**Drug/Combo**	**CI value**	**Dose SA**	**Dose EP**	**Dose VA**
SA		36.6956		
EP			153.796	
VA				37.0366

Data for Fa = 0.9

**Drug/Combo**	**CI value**	**Dose SA**	**Dose EP**	**Dose VA**
SA		307.684		
EP			2594.76	
VA				254.529

Data for Fa = 0.95

**Drug/Combo**	**CI value**	**Dose SA**	**Dose EP**	**Dose VA**
SA		1306.81		
EP			17731.2	
VA				944.257

Data for Fa = 0.97

**Drug/Combo**	**CI value**	**Dose SA**	**Dose EP**	**Dose VA**
SA		3656.99		
EP			69598.8	
VA				2399.92
